# Therapeutic administration of etoposide coincides with reduced systemic HMGB1 levels in macrophage activation syndrome

**DOI:** 10.1186/s10020-021-00308-0

**Published:** 2021-05-11

**Authors:** Karin Palmblad, Hanna Schierbeck, Erik Sundberg, Anna-Carin Horne, Helena Erlandsson Harris, Jan-Inge Henter, Ulf Andersson

**Affiliations:** 1 Department of Women’s and Children’s Health, Karolinska Institute at Karolinska University Hospital, 17176 Stockholm, Sweden; 2 Rheumatology Unit, Department of Medicine, Karolinska Institute at Karolinska University Hospital, 17176 Stockholm, Sweden; 3 Childhood Cancer Research Unit, Department of Women׳‬s and Children׳‬s Health, Karolinska Institute, 17177 Stockholm, Sweden; 4 Theme of Children, Karolinska University Hospital, 17176 Solna, Stockholm, Sweden

**Keywords:** HMGB1, Macrophage activation syndrome, HLH, FHL, Inflammation, Pathogenesis

## Abstract

**Background:**

Macrophage activation syndrome (MAS) is a potentially fatal complication of systemic inflammation. HMGB1 is a nuclear protein released extracellularly during proinflammatory lytic cell death or secreted by activated macrophages, NK cells, and additional cell types during infection or sterile injury. Extracellular HMGB1 orchestrates central events in inflammation as a prototype alarmin. TLR4 and the receptor for advanced glycation end products operate as key HMGB1 receptors to mediate inflammation.

**Methods:**

Standard ELISA and cytometric bead array-based methods were used to examine the kinetic pattern for systemic release of HMGB1, ferritin, IL-18, IFN-γ, and MCP-1 before and during treatment of four children with critical MAS. Three of the patients with severe underlying systemic rheumatic diseases were treated with biologics including tocilizumab or anakinra when MAS developed. All patients required intensive care therapy due to life-threatening illness. Add-on etoposide therapy was administered due to insufficient clinical response with standard treatment. Etoposide promotes apoptotic rather than proinflammatory lytic cell death, conceivably ameliorating subsequent systemic inflammation.

**Results:**

This therapeutic intervention brought disease control coinciding with a decline of the increased systemic HMGB1, IFN-γ, IL-18, and ferritin levels whereas MCP-1 levels evolved independently.

**Conclusion:**

Systemic HMGB1 levels in MAS have not been reported before. Our results suggest that the molecule is not merely a biomarker of inflammation, but most likely also contributes to the pathogenesis of MAS. These observations encourage further studies of HMGB1 antagonists. They also advocate therapeutic etoposide administration in severe MAS and provide a possible biological explanation for its mode of action.

## Introduction

This report replaces a study (Palmblad et al. 2015) that was retracted (Palmblad et al. 2020) because mass spectrometry data provided by one of the authors had been fabricated. The present report, where the incorrect data were removed, reaffirms the key information communicated at the time, and can be correctly cited as the first description of dynamically expressed systemic HMGB1 levels related to clinical courses during macrophage activation syndrome.

Macrophage activation syndrome (MAS) is a severe and potentially life-threatening complication of systemic inflammatory disorders (Ravelli et al. 2012). MAS typically appears in patients with systemic juvenile idiopathic arthritis (sJIA) and its adult equivalent, adult-onset Still’s disease (Ravelli et al. 2012); it is also reported in other inflammatory disorders including juvenile systemic lupus erythematosus (SLE) (Parodi et al. 2009) and Kawasaki’s disease (Simonini et al. 2010). Symptoms and signs of MAS include persistent fever, hepatosplenomegaly, lymphadenopathy, a reduction of all cellular blood elements, liver dysfunction, disseminated intravascular coagulation, and central nervous system dysfunction (Ravelli et al. 2012).

MAS expresses a close clinical resemblance to a group of histiocyte disorders collectively known as hemophagocytic lymphohistiocytosis (HLH). Hence, MAS is classified among the secondary (acquired) forms of HLH and is also called MAS-HLH (Emile et al. 2016). Secondary HLH most commonly occurs in response to an infection (often viral), or a malignancy or to autoimmune/autoinflammatory diseases (Ramos-Casals et al. 2014). Primary HLH is a genetic disorder of immune regulation caused by mutations in genes encoding proteins required for the cytolytic activity exerted by NK cells and cytotoxic T cells (Fischer et al. 2007). Impaired cytolytic capacity is also postulated as a key event in the pathogenesis of MAS, diminishing the ability to induce apoptosis needed for an immunologically silent elimination of target cells (Villanueva et al. 2005). Hence, cell death by other mechanisms, including necrosis and pyroptosis, will dominate in MAS and HLH leading to excessive activation and survival of macrophages, NK cells and T lymphocytes generating an overwhelming inflammatory reaction. A properly functioning cytotoxic defense system is needed to eliminate virally infected cells and cancer transformed cells, and to terminate immune reactions by killing activated autologous cells mediating inflammation.

HMGB1 is a ubiquitous nuclear protein with proinflammatory properties when released to the extracellular space, thus establishing HMGB1 as a prototypic damage-associated molecular pattern (DAMP) molecule or alarmin (Harris and Raucci 2006; Scaffidi et al. 2002). HMGB1 is passively leaked out of necrotic and pyroptotic cells (Harris and Raucci 2006; Lu et al. 2013). During apoptosis, HMGB1 will be terminally oxidized and strongly bound to the chromatin and retained in apoptotic bodies preventing HMGB1 from extracellular release (Bianchi and Manfredi 2004). The assembly of large multi-proteins complexes to activated inflammasomes generates active caspase-1, caspase-4, and caspase-5 formation, that control the release of IL-1α, IL-1β, IL-18, and HMGB1, and consequently result in a programmed proinflammatory cell death called pyroptosis (Lamkanfi et al. 2010; Lu et al. 2012). IL-1 and IL-18 are well-established and important mediators in MAS-HLH (Crayne et al. 2019; Ravelli et al. 2012; Schulert and Canna 2018; Weiss et al. 2018), while the presence and a functional role of HMGB1 in these conditions remain to be studied.

Extracellular HMGB1 generally exists in vivo linked to other molecules. Receptor usage causing inflammation depends on whether HMGB1 acts on its own or in complex with partner molecules. Macrophages expressing RAGE, but engineered to lack TLR4 expression, do not produce proinflammatory cytokines in response to an HMGB1 molecule without attached partners (Yang et al. 2010). The TLR4-MD-2 receptor complex is thus mandatory for the ability of uncomplexed HMGB1 to induce cytokine release. Extracellular HMGB1 binds many different extracellular proinflammatory molecules including histones, nucleosomes, DNA, RNA, SDF-1, IL-1α, IL-1β, and many pathogen-associated molecular pattern (PAMP) molecules including lipopolysaccharide (LPS) (Andersson et al. 2018; Deng et al. 2018). These complexes act in synergy via binding to RAGE expressed on activated macrophages and additional cells (Andersson et al. 2018; Deng et al. 2018). The HMGB1-RAGE interaction mediates a cellular import of the HMGB1-partner molecule complexes, but not an immediate subsequent intracellular signaling (Deng et al. 2018; Xu et al. 2014; Yuan et al. 2020). The endocytosed HMGB1-complexes are transported to the endolysosomal compartment. The important physiological function is to bring the HMGB1-transported extracellular danger molecules for lysosomal degradation. Many of these danger molecules are not appropriately recognized by endogenous antibodies with a capacity to deliver the molecules to the lysosomal compartment via cell surface-expressed Fc-receptors. However, the risk with the HMGB1-assisted intracellular transport is that a high intralysosomal HMGB1 concentration at acidic pH may disrupt the lysosomal membrane allowing the attached DAMPs and PAMPs access to cytosolic sensors including multiple inflammasome complexes (Deng et al. 2018; Porat et al. 2018; Yang et al. 2016). The leakage prevents intralysosomal degradation of the danger molecules and enables them to activate proinflammatory intracellular sensors, which they would not reach without HMGB1 assist.

The HMGB1 redox isoform is key when HMGB1 acts on its own as a pro-inflammatory mediator. The redox state of the three cysteines present in an HMGB1 molecule determines subsequent bioactivities. Nuclear HMGB1 in a quiescent cell is always in the fully reduced form with all three cysteines expressing thiol groups. The fully reduced HMGB1 released extracellularly forms a complex with the chemokine CXCL12 (SDF-1) and initiates enhanced chemotaxis via CXCR4, compared to CXCL12 acting alone (Schiraldi et al. 2012). Gentle HMGB1 oxidation generates a disulfide bond between Cys23 and Cys45, but preserves Cys106 in the reduced form. This modification converts extracellular HMGB1 to a potent activator of proinflammatory cytokine production via TLR4 receptor stimulation (Yang et al. 2012). Disulfide HMGB1 loses its capacity to activate TLR4 when it is either reduced or further oxidized. Valid quantitative assays to analyze total HMGB1 levels in clinical samples are readily available, while regrettably, there is a global lack of methods enabling quantification of HMGB1 redox isoforms.

We here aimed to study the presence and kinetic expression of HMGB1 in MAS, and to examine a possible functional connection between etoposide therapy and the role of HMGB1 in MAS-HLH.

## Materials and methods

### Patients

Four children aged 3 to 15 years, three previously diagnosed with systemic onset juvenile idiopathic arthritis (sJIA) and one with juvenile systemic lupus erythematosus (SLE), who all presented with MAS and fulfilled current MAS criteria were studied during a one-year-period from November 2010 to November 2011 (Table 1) (Parodi et al. 2009; Ravelli et al. 2005). The diagnosis of sJIA was made on the basis of the criteria of the International League of Associations for Rheumatology (Petty et al. 1998) and the SLE patient fulfilled the American College of Rheumatology (ACR) revised SLE criteria. MAS was diagnosed on the combination of clinical features, including cytopenia or sudden decrease in white blood cell counts and/or platelet counts, coagulopathy, and liver dysfunction, according to the guidelines proposed by Ravelli et al. and Parodi et al. (Parodi et al. 2009; Ravelli et al. 2005). Three patients also fulfilled diagnostic criteria for HLH according to the HLH-2004 criteria (Henter et al. 2007). All patients had markedly elevated inflammatory parameters, including CRP and ferritin, and needed intensive care treatment (Table 1). Three patients (patients 1, 2, and 4) expressed CNS involvement, which was very severe in two of them (patients 2 and 4). During the development of MAS, all four patients had been administered high corticosteroid doses, and, in addition cyclosporin A (CsA), hydroxychloroquine, etanercept and/or anakinra. At the time of developing MAS, therapy included tocilizumab and methotrexate; tocilizumab, methotrexate and prednisolone; anakinra, CsA and betamethasone; and hydoxychloroquine and prednisone; respectively. In the two patients with tocilizumab therapy, infections with Epstein–Barr virus infection and varicella-zoster virus infection preceded the development of MAS (Table 1). Due to the severe clinical MAS presentations including CNS affection in three patients and rapidly progressive pancytopenia in the fourth, and the similarities between MAS and HLH (Ramanan and Schneider 2003), we administered etoposide and corticosteroids which is standard therapy in HLH (Henter et al. 2007; Trottestam et al. 2011). However, since the treatment protocols HLH-94 and HLH-2004 were originally designed for infants with primary HLH (familial hemophagocytic lymphohistiocytosis, FHL) and are associated with a considerable risk of neutropenia and infections, we administered etoposide at lower doses (50–100 mg/m^2^) and less frequent intervals (typically scheduled once weekly) than suggested in the HLH protocols (150 mg/m^2^, initially twice weekly) (Horne et al. 2021). The duration of the etoposide treatment ranged from 6 to 10 weeks. All patients responded with dramatic improvement to the addition of etoposide therapy. The clinical characteristics and treatment regimens of the patients and treatment regimens are summarized in Table 1 (Horne et al. 2017).

**Table 1:** Clinical characteristics of the four children with MAS

	Patient 1	Patient 2	Patient 3	Patient 4
Sex	Female	Female	Male	Female
Age at onset of MAS	9 y	3 y	5 y	15 y
Underlying disease	sJIA	sJIA	sJIA	SLE
Ongoing treatment at onset of MAS	Tocilizumab, MTX	Oral steroids, tocilizumab, MTX	Oral steroids, anakinra, CsA	Oral steroids, hydroxy-chloroquine
Previous treatment	Oral steroids, MP pulses, etanercept	MP pulses, etanercept	Oral steroids, IVIG, CsA, anakinra, MP pulses	Oral steroids, hydroxy-chloroquine
Verified infections	EBV	VZV	None	UTI: *E Coli*
Diagnostic MAS criteria Diagnostic HLH-2004 criteria sCD25 (U/ml)	Yes Yes (6/8) > 7500	Yes Yes (5/8) > 7500	Yes No (3/8) 3309	Yes Yes (6/7) 3460
Neurological symptoms	Moderate	Severe	No	Severe
ICU-care	Yes	Yes	Yes	Yes
First-line MAS therapy	MP-pulses	MP-pulses	MP-pulses, anakinra 4 mg/kg	MP-pulses
Etoposide	100 mg/m^2^ × 3 150 mg/m^2^ × 5	100 mg/m^2^ × 9	50 mg/m^2^ × 2 100 mg/m^2^ × 7	50 mg/m^2^ × 3 75 mg/m^2^ × 2 100 mg/m^2^ × 2
Weeks on etoposide	9	8	10	6
Additional MAS-HLH treatment	Oral steroids, CsA, rituximab	Oral steroids	Oral steroids, CsA	Oral steroids, CsA plasmapheresis
Clinical response	Complete	Complete	Complete	Severe CNS sequele

*sJIA* systemic onset juvenile idiopathic arthritis; *SLE* systemic lupus erythematosus; *MTX* Methotrexate; *CsA* cyclosporine A; *oral steroids* oral corticosteroids; *MP-pulses* methylprednisolone pulses; *IVIG* intravenous immunoglobulins; *EBV* Epstein–Barr virus; *VZV* varicella zoster virus; *UTI* urinary tract infection; *sCD25* soluble interleukin-2 receptor; *ICU* intensive care unit; *MAS-HLH* Macrophage Activating Syndrome-Hemophagocytic lymphohistiocytosis; *CNS* central nervous system

Blood samples from patients with oligoarticular, polyarticular, enthesitis-related, or other types of JIA according to the ILAR criteria, as well as blood samples from 10 healthy pediatric controls (range 2–14 years), were collected at Astrid Lindgren Children’s Hospital and analyzed for comparison of total HMGB1 levels. This study was approved by the Ethics Committee, Karolinska Institutet in Stockholm, Sweden. Parents and patients gave informed consent before inclusion.

### Blood samples

Sera were obtained from blood samples collected in tubes without additives and plasma were collected in EDTA tubes. Blood samples were centrifuged within 1 h after sampling at 1440 g for 10 min and cells were removed and the supernatants were stored at −80 °C until assayed. We serially determined the systemic levels of inflammasome-associated HMGB1, IL-1α, IL-1β and IL-18 and additionally IFN-γ, MCP-1 and ferritin in the patients before, during and after therapeutic intervention until inflammation had resolved.

### ELISA assay for HMGB1 detection

HMGB1 levels were measured in undiluted plasma using HMGB1 ELISA kit II, according to the instructions of the manufacturer (IBL International, Germany). The lower limit for detection was 0.3 ng/mL.

### Cytometric bead array (CBA) and Bioplex for detection of IFN-γ, IL-1α, IL-1β, IL-18 and MCP-1

All sera were diluted 1:2 − 1:8 before analysis. IFN-γ, IL-1α, IL-1β, and IL-18 were measured by Bioplex (Bio-Rad Laboratories, Hercules, CA, USA) with a lower detection limit of 4–9 pg/mL. Levels of MCP-1 were measured by CBA using Human Soluble Protein Flex Sets and Human Soluble Protein Master Buffer Kits (B&D Biosciences, Pharmingen, San Diego, CA, USA) with a lower detection limit of 20 pg/mL. All analyses were performed according to the instructions of the manufacturers. Serum levels of ferritin were analyzed at the clinical laboratory at Karolinska University Hospital in Stockholm, Sweden.

### Statistical analysis

**Fig. 1:**
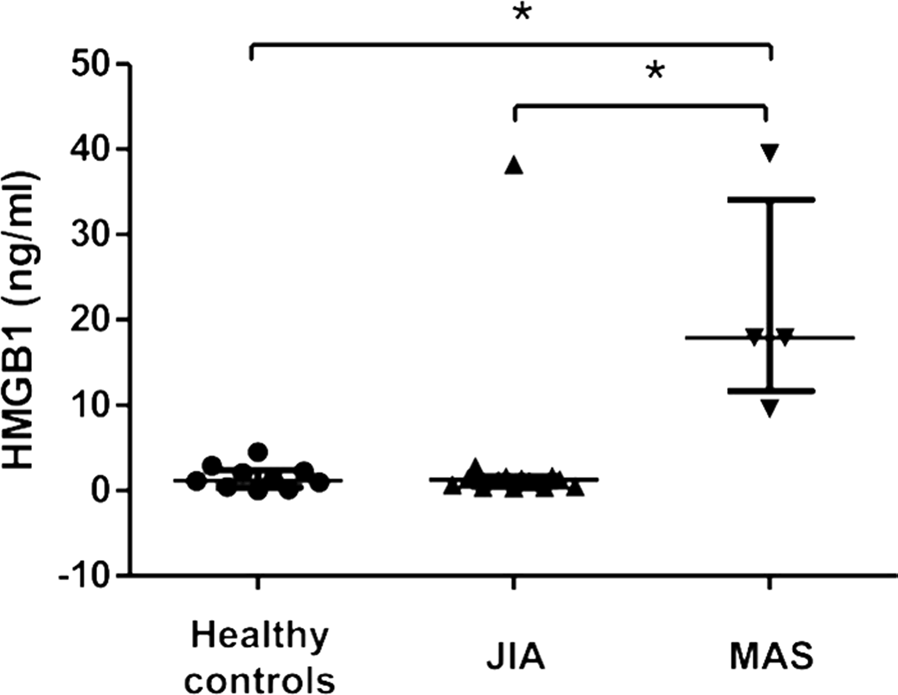
High systemic HMGB1 levels in MAS patients. Plasma HMGB1 levels measured by ELISA were markedly increased during severe MAS as compared to children with uncomplicated JIA and healthy pediatric controls. The HMGB1 levels in the same cohort of JIA patients and healthy control children have been published previously in (Schierbeck et al. 2013). *JIA* juvenile idiopathic arthritis, *MAS* macrophage activation syndrome. *p < 0.05

Data in Fig. 1 are presented as mean ± SEM. Differences between patient groups were determined by Student *t* test. Differences between plasma HMGB1 levels pre and post etoposide therapy were analyzed by the two-tailed, nonparametric Mann–Whitney U test. P values less than 0.05 were considered statistically significant.

## Results

Four children with exceptionally serious MAS (clinical data are outlined in Table 1) were studied retrospectively regarding the kinetic expression of systemic levels of HMGB1. First-line therapy based on high intravenous doses of corticosteroids, and anakinra (IL-1RA) in one patient, aimed to suppress the cytokine storm did not control the excessive inflammation in any of the studied MAS patients. Systemic etoposide treatment was subsequently administered based on its well-established efficacy in primary as well as secondary HLH disease. Special focus was directed on HMGB1 release in blood samples obtained closely before and after etoposide infusions, with the aim to evaluate whether etoposide would improve the inadequate ability to mediate apoptosis in critical target cells driving the uncontrolled inflammation. Lytic cell death leads to strong extracellular HMGB1 release, while apoptosis does not. Increased apoptosis would thus be expected to reduce extracellular HMGB1 levels. Studies of well-known proinflammatory mediators and biomarkers known to reflect the clinical course of MAS were conducted concurrently. These serum analyses included assessments of ferritin, IFN-γ, IL-1α, IL-1β, IL-18 and monocyte chemotactic protein (MCP-1).

**Fig. 2:**
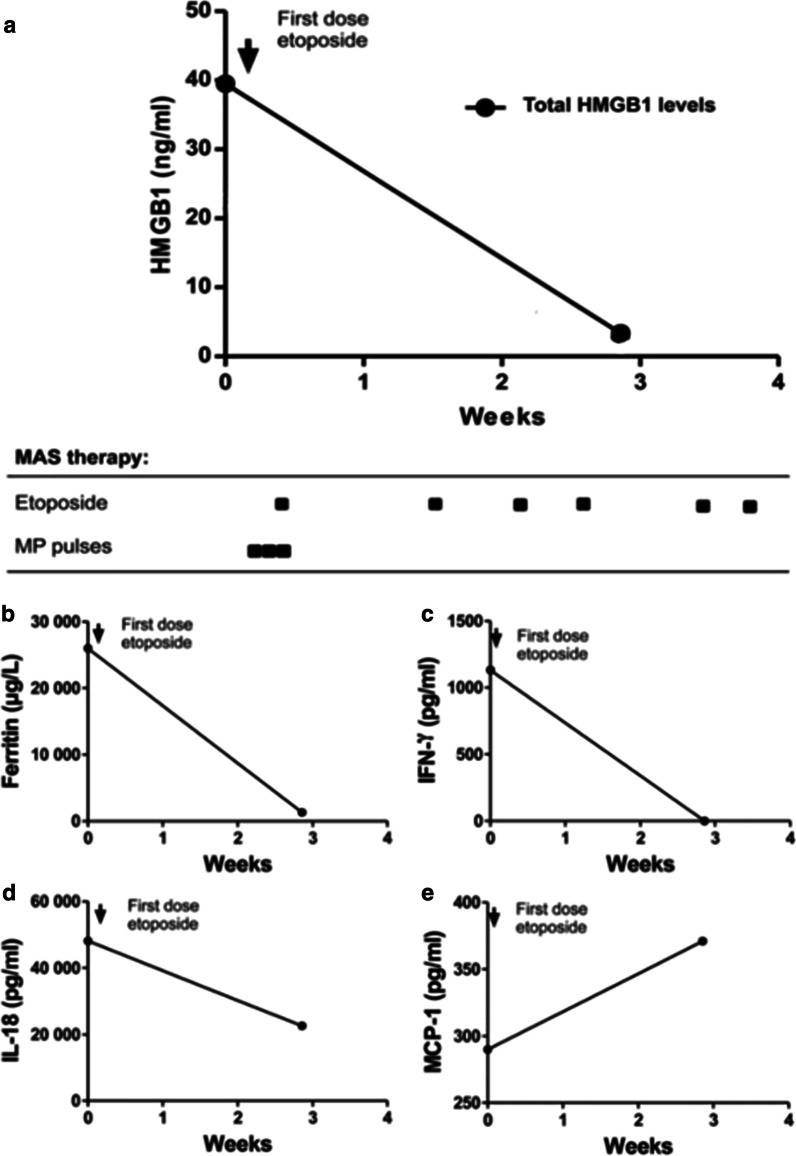
Longitudinal serum analyses before and after etoposide treatment in patient #1. High plasma levels of HMGB1 were observed during severe disease (**a**), and rapidly declined after initiation of etoposide treatment concomitantly with serum concentrations of ferritin (**b**), IFN-γ (**c**), and IL-18 (**d**). MCP-1 (**e**) levels peaked weeks later when the patient was recovering. *CsA* cyclosporine A; *MP-pulses* methylprednisolone pulses

**Fig. 3:**
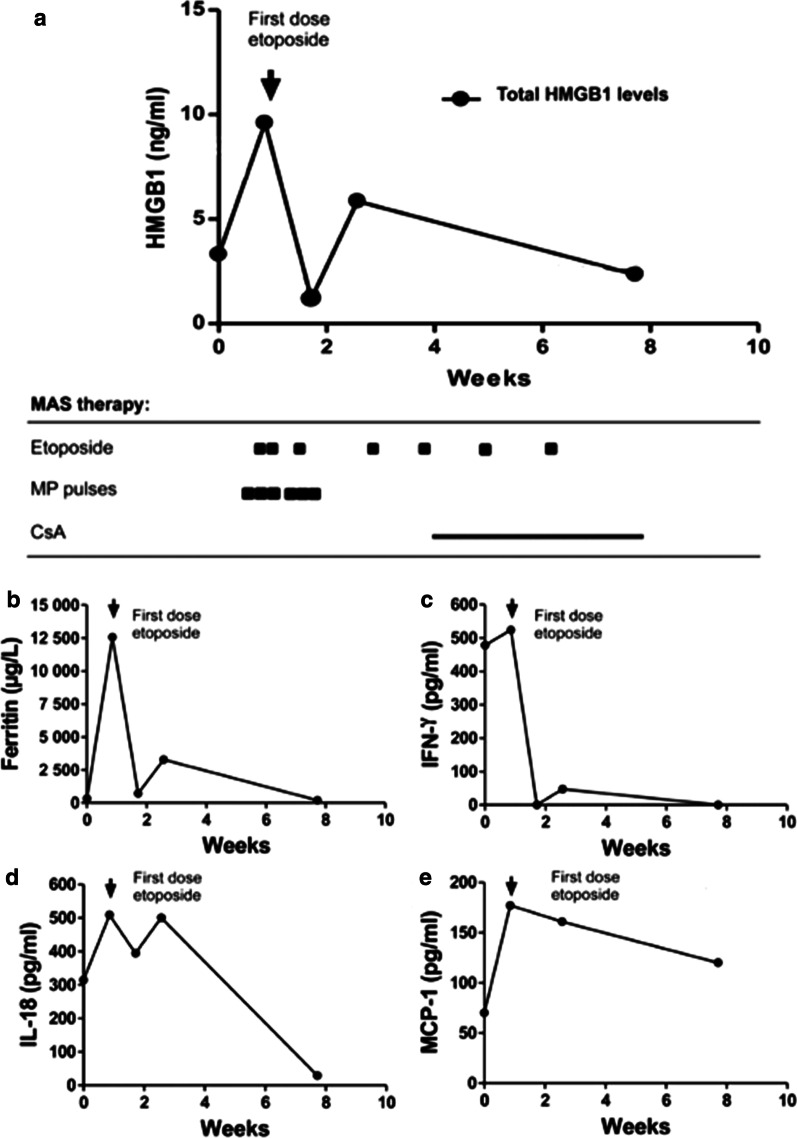
Serum analyses in patient #2 before and after treatment with etoposide. Two blood samples taken before and after etoposide therapy were analyzed when normalized levels of HMGB1 (**a**), ferritin (**b**) and IFN-γ (**c**) where documented after intervention with etoposide and subsequent clinical improvement. IL-18 declined but was still elevated (**d**) while MCP-1 increased (**e**). *CsA* cyclosporine A

**Fig. 4:**
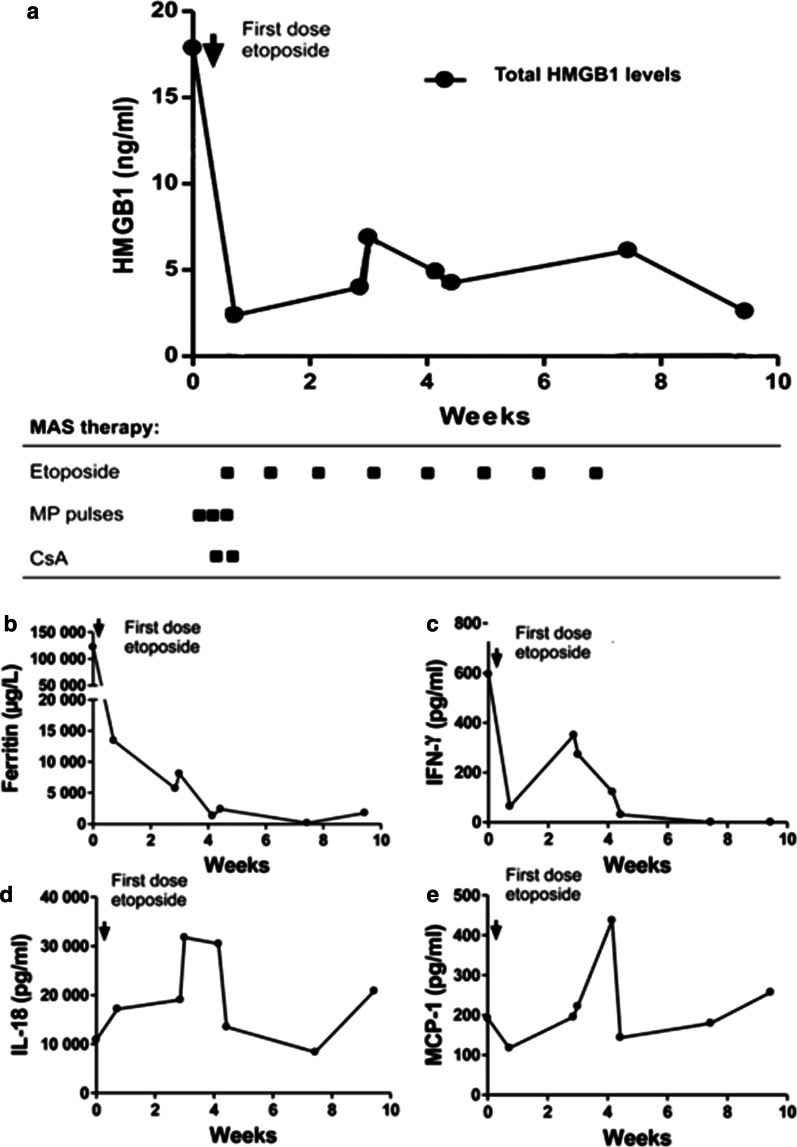
Longitudinal serum analyses before and after introduction of etoposide treatment in patient #4. The expression of plasma levels of HMGB1 (**a**), and serum levels of ferritin (**b**), IFN-γ (**c**), and IL-18 (**d**) corresponded very well to the clinical course of MAS with a rapid decline and clinical improvement after etoposide administration. MCP-1 levels were increased during the whole study period (**e**). *CsA* cyclosporine A; *MP-pulses* methylprednisolone pulses

**Fig. 5:**
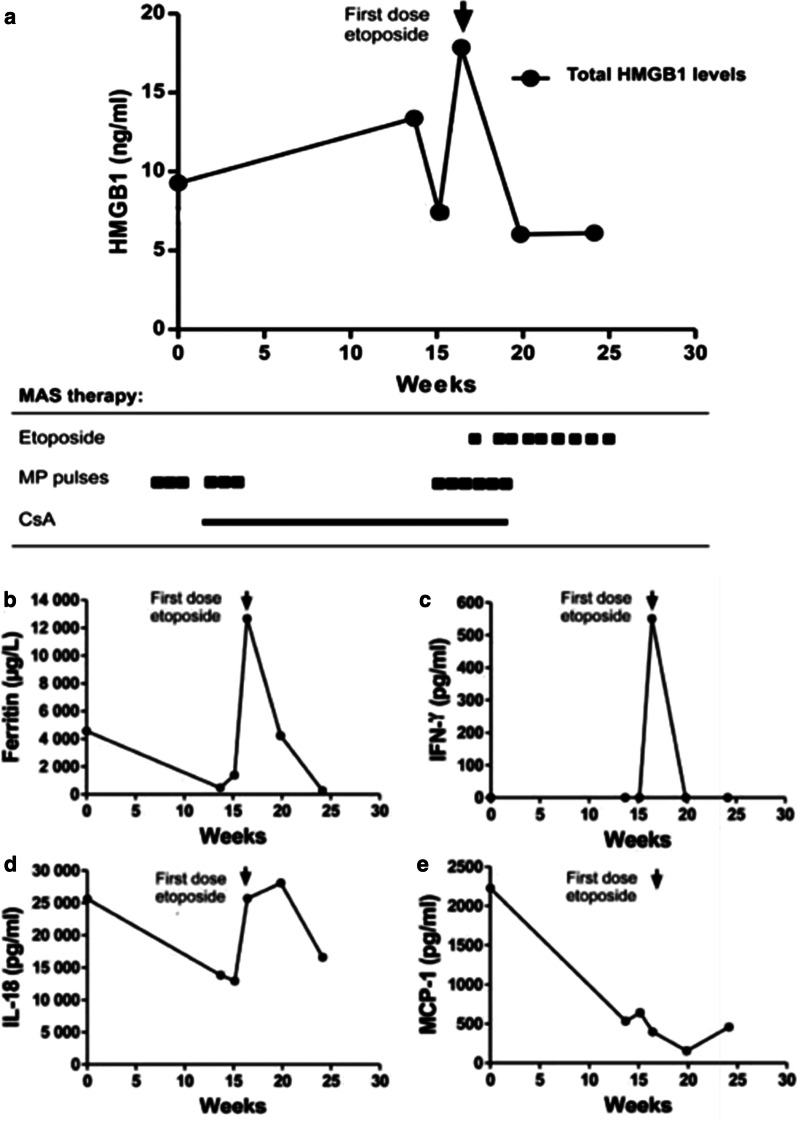
Longitudinal serum analyses before and after introduction of etoposide treatment in patient #3. The progression of plasma HMGB1 levels is illustrated in **a**. The first two plasma samples were collected at onset of sJIA without MAS manifestations. The HMGB1 levels increased at onset of MAS and declined promptly after treatment with etoposide infusions. Increased serum concentrations of ferritin (**b**) and IFN-γ (**c**) were documented during active MAS followed by a normalization post etoposide treatment. Serum IL-18 levels (**d**) were distinctly increased during the entire observation period with peak values during active phases of MAS. Serum MCP-1 levels did not reflect the clinical course (**e**). CsA: cyclosporine A; MP-pulses: methylprednisolone pulses

Total plasma HMGB1 levels in MAS patients before initiation of etoposide were significantly higher (p < 0.05) compared to those observed in JIA patients without MAS and healthy children (Fig. 1). The peak systemic HMGB1 levels were recorded at the same time as the MAS patients expressed maximal symptoms and signs (Figs. 2, 3, 4 and 5a). Control of inflammation with a clinical stabilization in the patients coincided with the initiation of add-on etoposide administration, when a prompt decrease of the systemic HMGB1 levels ensued.

Ferritin is a protein that stores iron in a soluble form and its synthesis is regulated by intracellular iron, inflammatory cytokines and oxidative stress. Serum ferritin levels are exceptionally increased in HLH and MAS. Hemophagocytosis results in enhanced uptake of haptoglobin-hemoglobin complexes by macrophages triggering a production of ferritin to sequester the excessive amount of free iron. Serum ferritin levels are used as the golden standard parameter to monitor the clinical course of HLH and MAS-HLH. All our studied MAS patients expressed serum ferritin levels that followed a parallel temporal course to that of plasma HMGB1 levels (Figs. 2, 3, 4 and 5a) and all these parameters mirrored the clinical course with decreased levels after etoposide administration coinciding with clinical improvement (Figs. 2, 3, 4 and 5b). Especially patient #3 demonstrated a dramatic decline in serum ferritin levels from 121 937 to 13 416 µg/L (upper normal range should be below 35 µg/L) within a few days after etoposide infusions (Fig. 4b).

IFN-γ is the key macrophage-activating cytokine and is released from activated NK cells and T lymphocytes. IFN-γ primes the capacity of macrophages for phagocytosis and for proinflammatory cytokine production and is thus a central cytokine in the pathogenesis of HLH and MAS (Henter et al. 1991; Ravelli et al. 2012; Zoller et al. 2011). Serum levels of IFN-γ were markedly increased during severe disease periods in all studied MAS patients and these levels promptly decreased during clinical resolution after etoposide administration (Figs. 2, 3, 4 and 5 panel C).

IL-1α, IL-1β, and IL-18 are also central pathogenic mediators in MAS (Miettunen et al. 2011; Shigemura et al. 2011) and all these three molecules are released during pyroptosis, which likewise is an important pathway for HMGB1 release (Lamkanfi et al. 2010; Lu et al. 2012). IL-18 is a potent inducer of IFN-γ production. Markedly increased serum levels of IL-18 were documented in all our patients, with peak values appearing somewhat later than systemic HMGB1, IFN-γ and ferritin levels (Figs. 2, 3, 4 and 5d). Serum IL-1α and IL-1β were not detected at any time point in any of the studied MAS patients (data not shown). IL-1 exerts potent biological effects at the low pg/mL range, and we question whether our detection methods were sensitive enough to detect these low levels. The chemokine MCP-1 is increased in JIA patients (Schierbeck et al. 2013) and has been implicated in the pathogenesis of HLH, where serum concentrations correlate well to disease activity (Tamura et al. 2008). In the present study, serum MCP-1 levels did not change in any consistent manner in response to treatment and the levels peaked around 3–4 weeks after the initiation of therapy (Figs. 2, 3, 4 and 5 e).

**Fig. 6:**
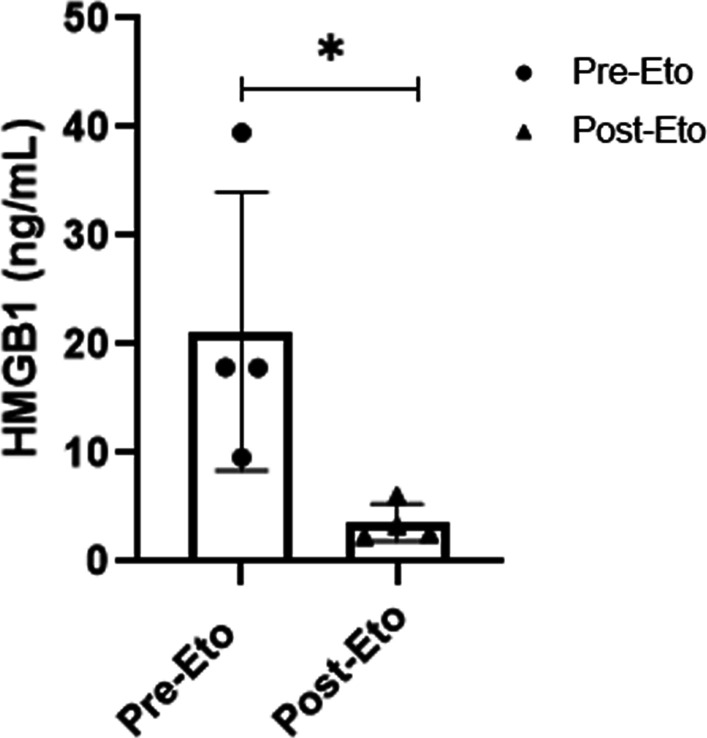
Plasma HMGB1 levels in the four MAS patients before and after etoposide therapy. Plasma HMGB1 measured by ELISA was markedly increased in all patients during severe MAS before etoposide (Eto) treatment was added to ongoing therapy. Plasma HMGB1 concentration immediately before first dose of etoposide was compared to last available sample. HMGB1 levels were significantly reduced (*p < 0.05) post etoposide administration

To summarize, the results were consistent in all four MAS patients with high levels of plasma HMGB1, promptly declining during clinical resolution coinciding with therapeutic etoposide therapeutic intervention (Fig. 6). The dynamic shift of systemic levels of IFN-γ, IL-18, and ferritin concurred roughly with those for HMGB1 and the clinical courses, whereas MCP-1 levels did not.

## Discussion

We here present four children with severe MAS monitored with serial measurements of selected proinflammatory mediators present systemically. We observed markedly elevated plasma HMGB1 levels during active MAS in all patients, which is an original observation not reported before. Disease control was achieved in all patients when etoposide administration was added to the conventional anti-inflammatory treatment, with a marked concomitant decline of the extracellular HMGB1 levels.

Etoposide is a chemotherapeutic drug that inhibits topoisomerase II and the subsequently induced dysregulation generates DNA synthesis errors resulting in apoptotic cell death in rapidly dividing or activated cells. It has been demonstrated in an animal HLH model, based on lymphocytic choriomeningitis virus infection in perforin-deficient mice, that etoposide selectively ablated activated, pathogenic T cells via induction of apoptotic cell death. Inactive T cells, including memory T cells, were not influenced by the treatment (Johnson et al. 2014). Etoposide is the mainstay of treatment for primary HLH patients (Bergsten et al. 2017), but has not yet undergone controlled trials for MAS patients. The life-threatening courses of MAS in our patients despite regular therapy prompted us to administer a supplementary treatment. Since the clinical expressions of HLH and MAS are indistinguishable, we chose etoposide but at a reduced dose as compared to what is commonly used for primary HLH. A considerable risk regarding the use of etoposide is elimination of too many immunocompetent cells needed for microbial defense, as in patient #4, who developed neutropenia and bacterial sepsis after the etoposide infusion. Of note, the neutrophil count in this patient had already declined to 0.3 × 10^9^/L before etoposide therapy was initiated. Prior to the onset of MAS-HLH, all patients had received treatment with corticosteroids and at least one more immunomodulatory drug (CsA, hydroxychloroquine, etanercept, IVIG, and/or anakinra) (Table 1). At the diagnosis of MAS-HLH, all three patients with sJIA were administered interleukin blockade with tocilizumab (n = 2) or anakinra (n = 1) in combination with at least one additional immunomodulatory drug (methotrexate or CsA), while patient #4 with lupus received hydroxychloroquine and prednisone (Table 1). Administration of etoposide was followed by drastically reduced HMGB1 levels, and clinical improvement. Whether the observed outcome parameters were due to effects generated by etoposide alone or etoposide acting synergistically with other therapeutic agents cannot be resolved in our pilot study.

Significant amounts of extracellular HMGB1 may be discharged either by secretion from activated immune cells or by passive release from necrotic, pyroptotic or damaged cells, but not from apoptotic cells (Kang et al. 2014). How could then extracellular HMGB1 contribute to the systemic inflammation in MAS? There are several HMGB1-dependent pathways that may be involved if we postulate that the initiating event in MAS in a given sterile or infectious insult is an impaired capacity to provide key cytolytic molecules needed for induction of apoptosis in critical target cells. Other modes of cell deaths such as necrosis or pyroptosis will then take place instead. These lytic cellular events generate extracellular HMGB1 release driving inflammation via chemotactic signals via CXCR4 (Schiraldi et al. 2012) and activating recruited inflammatory cells via TLR4/MD-2 (Kim et al. 2013; Yang et al. 2010) to produce proinflammatory cytokines and to induce powerful phagocytic responses. A deficient cytolytic capacity to eliminate activated NK cells and cytotoxic T cells, which are key producer cells of IFN−γ, will further contribute to the macrophage activation in the patients. The almost identical temporal changes observed in our patients regarding systemic levels of IFN−γ and HMGB1 in response to therapy are in line with this assumption (Figs. 2, 3, 4 and 5). It is important to consider that it is most likely only the cytolytic pathway that is functionally compromised in cytotoxic NK cells and CD8-positive T cells in HLH and MAS-HLH patients. When these cells get activated they will produce large amounts of IFN-γ and other proinflammatory mediators. Furthermore, a poor cytolytic activity in HLH and MAS-HLH patients may lead to a failure to kill autologous virus-infected cells and thus the source for antigen stimulation will persist leading to long-lasting antigen-driven activation of the immune system escalating the inflammatory response.

Extracellular HMGB1 activates proinflammatory cytokine formation via two different receptor systems. The disulfide HMGB1 isoform activates TLR4 in an analogous way to LPS (Yang et al. 2015). Furthermore, any redox form of extracellular HMGB1 avidly complex-binds many different extracellular danger-associated molecular pattern (DAMP) molecules and pathogen-associated molecular pattern (PAMP) molecules abundantly released during cell death and infectious processes during severe MAS. These HMGB1-DAMP/PAMP complexes bind to RAGE expressed on activated macrophages and get endocytosed to the endolysosomal system and may end up in the cytosol via mechanisms outlined in "Introduction section. The HMGB1-imported DAMPs and PAMPs may thus cause inflammasome activation in the cytosol generating a cytokine storm, coagulopathy, and pyroptosis (Deng et al. 2018; Yang et al. 2019). The prototypical inflammatory mediators released after inflammasome activation and pyroptosis are IL-1α, IL-1β, IL-18 and HMGB1. An attractive therapeutic strategy to prevent these dangerous events would thus be to convert pyroptosis to apoptosis, when the intracellular components are retained within cellular membranes never to reach the extracellular space. The reduction of systemic levels of HMGB1 and IL-18 in the patients after repeated etoposide administration supports the notion that etoposide succeeded to prevent excessive pyroptosis indicated by reduced extracellular levels of mediators released during pyroptosis. However, systemic IL-1α and IL-1β, which are also released during pyroptosis, were never detected in the serum of any of the four patients. We speculate that our detection methods were not sensitive enough to monitor IL-1 release.

Severe forms of COVID-19 share many clinical and laboratory features with MAS. The life-threatening inflammation in severe COVID-19 is likewise sustained by a cytokine storm and strongly increased systemic levels of ferritin, LDH and HMGB1 represent additional common denominators (Colafrancesco et al. 2020; Retamozo et al. 2021). Extracellular LDH and HMGB1 are consequences of lytic cell death occurring particularly in respiratory epithelial cells in severe SARS-CoV-2 infection. Active release from stimulated macrophages and other innate immunity cells further contributes to the strongly increased plasma HMGB1 levels present in severe COVID-19 patients (Chen et al. 2020). Corticosteroid therapy ameliorates outcomes in both COVID-19 and MAS (Horby et al. 2021; Trottestam et al. 2011).

HMGB1 is hypothetically a future target molecule of interest for MAS therapy. However, the fact that extracellular HMGB1 generally transports complex-bound partner molecules sets hurdles to the development of HMGB1-specific antagonistic molecules. However, one such candidate molecule that binds HMGB1 during inflammation is CD24, a cell surface protein normally expressed on hematopoietic cells. HMGB1-CD24 complexes form tri-molecular complexes with Siglec-10, another receptor on these cells. The functional outcome of this interaction is a potent downregulation of critical intracellular signal pathways needed for multiple central inflammatory mechanisms (Chen et al. 2009; Tian et al. 2020). There are ongoing therapeutic phase II/III studies in severe COVID-19 patients based on recombinant CD24 molecules administered systemically or via inhalation. The future results might help to resolve whether these therapeutic approaches should warrant treatment studies in MAS.

Our present study has a proof-of-concept design and involves only four MAS-HLH patients. Thus, the results need to be replicated in extended studies to conclude on causative events. Nevertheless, we noted a distinct temporal relationship between initiation of supplementary therapeutic etoposide administration and clinical improvement coinciding with a decline of systemic HMGB1, IL-18, IFN-γ, and ferritin levels.

## Conclusions

Systemic HMGB1 levels in MAS are here reported for the first time. The results suggest that HMGB1 is not merely a biomarker of inflammation but might also contribute to the pathogenesis of MAS. Our observations encourage further studies of therapeutic etoposide administration in severe MAS and provide a possible biological explanation for its mode of action. Etoposide converts lytic to apoptotic cell death and thereby prevents passive cellular HMGB1 release.

## Data Availability

The data underlying this article cannot be shared publicly due to regulations under the Swedish law. According to the Swedish Ethical Review Act, the General Data Protection Regulation, the Public Access to Information and Secrecy Act, data can only be made available, after legal review, for researchers who meet the criteria for access to this type of confidential data where patient identity risks being revealed. Requests regarding the data may be made to the corresponding author.
